# Proceedings of the 2012 MidSouth computational biology and bioinformatics society (MCBIOS) conference

**DOI:** 10.1186/1471-2105-13-S15-S1

**Published:** 2012-09-11

**Authors:** Jonathan D Wren, Mikhail G Dozmorov, Dennis Burian, Rakesh Kaundal, Susan Bridges, Doris M Kupfer

**Affiliations:** 1 Arthritis and Immunology Research Program, Oklahoma Medical Research Foundation; 825 N.E. 13th Street, Oklahoma City, OK 73104-5005, USA; 2 Biochemistry and Molecular Biology Dept, Univ of Okla Health Sciences Center, USA; 3 Civil Aerospace Medical Institute, Federal Aviation Administration, Oklahoma City, OK 73169, USA; 4 National Institute for Microbial Forensics & Food and Agricultural Biosecurity, Department of Biochemistry & Molecular Biology, Oklahoma State University, Stillwater, OK 74078, USA; 5 Department of Computer Science and Engineering, Mississippi State University, Box 9637, Mississippi State, MS 39762, USA

**Keywords:** bioinformatics, conferences, MCBIOS, ISCB

## Introduction

The ninth annual conference of the MidSouth Computational Biology and Bioinformatics Society (MCBIOS 2012), "Making Sense of the Omics Data Deluge", took place in Oxford, Mississippi February 17-8 2012. This year's Conference Chairs were Dr. Dawn Wilkins, of the University of Mississippi and Dr. Doris Kupfer, also the current MCBIOS President (2011-2), from the Federal Aviation Administration. There were 170 registrants and a total of 106 abstracts (34 oral presentations and 72 poster session abstracts).

Keynote speakers for 2012 were Dr. Michael Gribskov, Purdue University, who gave the opening address, "After the Deluge: Bioinformatics meets big data"; Dr. David J. States, OncProTech LLC, who gave his presentation remotely via WebEx entitled "Data Intensive Proteomics"; and Sultan Meghji, Appistry Inc. presenting the Saturday morning address, entitled "Simple, Fast and Affordable - Turning the myriad of data into action - technologies to support personalized medicine" and invited speaker, Dr. William Slikker, Director of the Food and Drug Administration's, National Center for Toxicological Research, presented a talk entitled "Regulatory Science: Challenges and Progress" outlining the role research at the FDA plays in their regulatory responsibilities.

Participants also had the opportunity to attend hands-on workshops on NCBI tools, presented by Dr. Peter Cooper, NCBI/NLM/NIH staff scientist, and a collaboration workshop focused on the timber rattlesnake genome, facilitated by Dr. Ed Perkins, Army Corp of Engineers.

The winners of conference awards were:

Best Oral Presentations (students):

1st Place: Shana Stoddard, University of Mississippi

2nd Place: Neal Platt, Mississippi State

3rd Place: Aleksandra Markovets, UALR

Best Oral Presentations (Post-Doctoral fellows):

1st Place: Mikhail Dozmorov, OMRF

2nd Place: Zhichao Liu, NCTR

Best Poster (Computation):

1st Place: Shraddha Thakkar, UAMS

2nd Place: Xingyan Kuang, University of Missouri

3rd Place: Sule Dogan, Mississippi State

Best Poster (Biology):

1st Place: Tamer Aldwairi, Mississippi State

2nd Place: Dilip Gautam, Mississippi State

3rd Place: Bin Pang, University of Missouri

## Proceedings summary

This year, there were 13 papers accepted for publication in the conference proceedings [1-13] out of a total of 20 submitted (65%), which was the lowest number of papers published since the first MCBIOS conference in 2003 which also accepted 13 papers. It was the second lowest number of papers submitted to the proceedings (17 were submitted in 2004). This was a substantial drop from the 21 papers published in last year's Proceedings [14-34]. All papers were peer-reviewed by 2 or more reviewers. Our goal is to be inclusive, yet rigorous in the peer-review process such that accepted papers are both high quality and reflective of the work presented at the conference. Papers generally fell into five categories:

## Genomic analysis

Ptitsyn *et al *report an algorithm for analysis of whole genomes in terms of the genes that they share. It provides an important way to quantify gain and loss of genes across phyla, and the authors have identified core genes that are common to each phylum [6].

Verma and Melcher [8] describe a Support Vector Machine (SVM) model for distinguishing peptides originating from plant host proteins or from proteobacterial plant pathogen proteins. A feature set consisting of a combination of both single amino acid compostions and dipeptide compositions exhibited the highest accuracy.

Yao *et al. *[9] present a detailed phylogenetic and transcriptome analysis of three classes of the secondary-wall-associated NAC domain transcription factors across 19 higher plant species. In addition, computational modeling is used to predict the genes regulated or co-regulated by these transcription factors. The study reveals coordinative functioning of several NAC genes and a number of novel genes and pathways that can potentially be involved in biosynthesis of cell walls.

## Systems biology/pathways

Abundance of different databases literally creates an "omics data deluge". Hui Huang et al [3] addressed this issue by creating PAGED database http://bio.informatics.iupui.edu/PAGED, that include data from OMIM, GAD, MSigDb, miRecords and other databases as a one stop solution for exploratory science.

Zhang and Drabier [10] used different aspects of data integration by compiling an Integrated Pathway Analysis Database (IPAD, http://bioinfo.hsc.unt.edu/ipad). This database defines associations between genes, proteins, pathways, diseases, drugs and organs, essential in understanding the relationships between these entities. These relationships can be quantified by running enrichment analysis with flexible threshold options.

Zhang and Berleant [12] developed a Java application BirdsEyeView http://metnetdb.org/MetNet_BirdsEyeView.htm for visualizing gene lists and expression data in context of cellular localization, pathways, and gene ontology annotations. Developed for plant research, this customizable tool provides flexible and intuitive understanding of the processes and pathways affected by the genes of interest.

## RNA-seq

Reddy *et al. *used RNA-Seq to develop an expression based data analysis workflow using freely available software to validate and expand the existing annotation of the cattle pathogen, *Mannheimia haemolytica *PHL213 [7]. Using the pipeline, the study confirmed existing *M. haemolytica *annotation as well as identified potential novel genes and operon structures, demonstrating and validating the use of this elegant, simple, and easily implemented bioinformatics pipeline.

## Proteomics

Zhang *et al *compare the effects of organelle enrichment on sensitivity of protein identification by high-throughput mass-spec from aerials of *A. thaliana*, and further compare and contrast the biological effects of two hormone treatments, Zeatin and brassinosteroid, on protein expression levels in mitochondria and chloroplasts. Their results suggest that physical enrichment of organelles increases the sensitivity of the assay to identify organelle specific proteins. In addition, they find that the two hormones affect different biological pathways to achieve a similar physiological effect, an increase in biomass for bioenergy production [13].

Zhang and Su analyzed the flexibility of protein structures using different structures of identical proteins based on structural comparison, secondary structure and sequence alignment, and report that proteins have several stable conformations, and that structures for the identical sequences may significantly differ from one another [11]. This will be helpful in evaluating the accuracy of protein structure prediction methods, e.g. one may need to employ molecular dynamic simulation to construct a structure set as criteria for such studies.

Pechan and Gwaltney investigated the relationship between tandem mass spectral fragment ion intensities and the distribution of *in vacuo *protonation states that can be modeled from peptide sequences [5]. Their work suggests that it is possible to calculate the ion intensities in the mass spectra of peptides, based solely on the protein's amino acid sequence.

## Miscellaneous

Halil Bisgin *et al *use topic modeling to analyze pharmacological similarity and evaluate their system in terms of its potential to reposition drugs - that is, to find additional uses for them. Doing so is important because new drug development is extremely expensive and time-consuming [1].

Zhifa Liu *et al *evaluated four different Bayesian network scoring functions, Minimum Description Length (MDL), Akaike's Information Criterion (AIC), Bayesian Dirichlet equivalence score (BDeu) and factorized Normalized Maximum Likelihood (fNML), and analyzed their performance in terms of success rate on recovering 'true' gold standard networks [4]. They report that MDL outperforms other scoring functions. This study would provide useful information when analyzing biological networks, such as the gene regulatory networks (GRN).

Fu et al [2] applied multiple instance learning via embedded selection (MILES) for the construction of quantitative structure-activity relationship (QSAR) between 3D shapes of a bioactive compounds with their targets. The authors demonstrated that their method, built solidly on previous research, allows better drug activity prediction without overfitting.

## Future meetings

The Stoney Creek Inn & Conference Center in Columbia, Missouri will be the site of MCBIOS 2013 to be held April 5-6. This will be the tenth anniversary of the MCBIOS conference and will be entitled "The 10th Anniversary in a Decade of Change: Discovery in a Sea of Data". The 2012-2013 MCBIOS President is Ed Perkins, US Army Engineer Research and Development Center and Andy Perkins, Mississippi State University, is now the President-elect. MCBIOS is a regional affiliate of the International Society for Computational Biology http://www.ISCB.org. For information regarding MCBIOS and our future meetings, see http://www.MCBIOS.org.

## Competing interests

The authors declare that they have no competing interests.

## Authors' contributions

All authors served as editors for these proceedings, with JDW serving as Senior Editor. All authors helped write this editorial.
